# Lessons from Beta-Thalassemia for Improving Iron Overload Monitoring and Management in Kidney Failure

**DOI:** 10.7759/cureus.77620

**Published:** 2025-01-18

**Authors:** Abdulqadir J Nashwan, Mohamed Yassin

**Affiliations:** 1 Nursing & Midwifery Research Department, Hamad Medical Corporation, Doha, QAT; 2 Department of Public Health, College of Health Sciences, QU Health, Qatar University, Doha, QAT; 3 Department of Hematology, National Centre for Cancer Care and Research, Hamad Medical Corporation, Doha, QAT

**Keywords:** beta-thalassemia, chelation therapy, chronic kidney disease, end-stage renal disease, ferritin, iron overload, kidney failure

## Abstract

Iron overload is a well-recognized complication in patients suffering from beta-thalassemia major (BTM) and end-stage renal disease (ESRD), recently known as kidney failure (KF), particularly in those who receive frequent blood transfusions or long-term iron supplementation. The mechanisms leading to iron overload differ slightly between these two conditions, but the management principles share considerable overlap. Lessons learned from monitoring and managing iron overload in patients with BTM can provide valuable insights into managing iron overload in patients with KF. This narrative review explores the parallels between these two conditions concerning iron overload, emphasizing the importance of early detection, personalized therapy, multidisciplinary care, patient education, and preventive strategies.

## Introduction and background

The prevalence of iron overload in patients with beta-thalassemia major (BTM) and end-stage renal disease (ESRD) or kidney failure (KF) who are undergoing hemodialysis (HD) or peritoneal dialysis (PD) presents distinct yet comparable patterns. In BTM, iron overload is nearly universal due to the need for frequent blood transfusions, leading to significant iron deposition in vital organs like the liver and heart. Without effective chelation therapy, more than 75% of BTM patients can experience severe cardiac and hepatic iron overload, contributing to heart failure and liver cirrhosis [1,2]. In contrast, patients with KF who are undergoing HD or PD may also develop iron overload, particularly those receiving high doses of intravenous iron or frequent blood transfusions to manage anemia. However, the prevalence of clinically significant iron overload in KF is lower compared to BTM, though hepatic iron accumulation is typical and can vary from 10-75% of KF patients on long-term dialysis, with cardiac iron overload being less frequent but still a concern in cases of excessive iron administration [3,4]. Both conditions highlight the importance of monitoring and managing iron overload to prevent organ-specific complications. Thus, this narrative review aims to explore the similarities and differences between these two conditions concerning iron overload, emphasizing the importance of early detection, personalized therapy, multidisciplinary care, patient education, and preventive strategies.

## Review

Iron overload in beta-thalassemia major

Patients with BTM, a genetic disorder characterized by ineffective erythropoiesis and lifelong transfusion dependence, are highly susceptible to iron overload [5]. Each unit of transfused blood contains approximately 200-250 mg of iron, which, over time, leads to an excess of iron in the body that cannot be excreted naturally [6]. This iron accumulates in vital organs such as the liver, heart, and endocrine glands, causing serious complications such as liver cirrhosis, heart failure, diabetes, and other endocrine dysfunctions [7,8].

The management of iron overload in BTM patients has evolved significantly over the years. One of the most important breakthroughs was the development of chelation therapy, which helps remove excess iron from the body. The availability of oral chelators like deferasirox has improved patient compliance and outcomes compared to earlier injectable options [1]. Additionally, non-invasive techniques for monitoring iron levels in organs, such as MRI T2* for cardiac iron and Relaxation rate (R2) MRI for hepatic iron, have allowed for more accurate assessments of iron burden [9].

Iron overload in kidney failure

Iron overload in KF primarily occurs in patients who receive frequent blood transfusions for anemia or high doses of intravenous iron as part of their anemia management strategy [10]. While iron deficiency is common in KF due to impaired erythropoiesis, excessive correction of this deficiency can lead to iron overload [11]. KF patients often receive erythropoiesis-stimulating agents (ESAs), which may increase iron demands and complicate management [12].

Iron overload in KF can result in oxidative stress, inflammation, and organ damage, especially in the liver and heart [13]. A standardized KF iron overload monitoring protocol complicates management [14]. Current practices often rely on serum ferritin and transferrin saturation (TSAT) levels. However, these markers can be unreliable due to their sensitivity to inflammation, which is common in KF patients [14]. Thus, lessons from BTM regarding accurate monitoring, personalized therapy, and early intervention are crucial for improving KF patients' iron overload outcomes (Table 1).

**Table 1:** Key lessons from BTM and their applications to KF BTM: Beta-Thalassemia Major, KF: Kidney Failure, MRI: Magnetic Resonance Imaging, TSAT: Transferrin Saturation, R2 MRI: Relaxation rate MRI (used to measure iron overload)

Aspect	Lessons from BTM	Applications to KF
Early Detection	Use of MRI T2* and R2 MRI for non-invasive monitoring of organ-specific iron overload.	Implement non-invasive monitoring techniques to supplement serum ferritin and TSAT.
Personalized Therapy	Tailored chelation therapy based on iron burden and patient tolerance.	Personalize iron supplementation and consider chelation in selected cases of transfusion-related overload.
Multidisciplinary Care	Collaborative care involving hematologists, cardiologists, and hepatologists.	Establish a multidisciplinary team, including nephrologists, cardiologists, and hematologists.
Minimizing Iron Intake	Limiting transfusion frequency and optimizing chelation therapy.	Adjust iron doses based on individual needs and minimize unnecessary transfusions or supplementation.
Patient Education	Educating patients about adherence to chelation therapy and regular monitoring.	Educate patients about the risks of iron overload and the importance of treatment adherence.

Early detection and monitoring

One of the key lessons from managing iron overload in BTM is the critical role of early detection and regular monitoring. In BTM, serum ferritin levels are commonly used as an initial screening tool for iron overload, though this marker has limitations in reflecting tissue iron levels [15,16]. The development of non-invasive imaging techniques, such as MRI T2* for cardiac iron and liver iron concentration (LIC) measured by R2 MRI, has revolutionized iron monitoring in BTM patients, allowing for more precise assessments of organ-specific iron overload [9,17].

In KF, the reliance on serum ferritin and TSAT for iron monitoring is inadequate. Inflammation often influences these markers, making it difficult to differentiate between true iron overload and a reactive rise in ferritin due to inflammatory processes common in KF [11,14]. Adopting non-invasive imaging techniques from BTM management, such as MRI T2* for heart iron monitoring and R2 MRI for liver iron, could significantly enhance the ability to detect iron overload early in KF patients [18]. For instance, a case series by Grant et al. involving five dialysis patients assessed LIC using MRI and compared these values to serum ferritin levels [19]. The findings revealed that serum ferritin consistently underestimated LIC, indicating significant iron overload despite seemingly acceptable ferritin levels. This suggests that relying solely on serum ferritin could lead to unrecognized iron accumulation. The study recommends incorporating MRI-based LIC assessments into routine practice to enhance iron management in dialysis patients. However, in countries where MRI is not readily available, relying on serum ferritin and TSAT as initial screening tools remains a more feasible option. We suggest that MRI be reserved for cases where these markers indicate significant iron overload or where organ-specific damage is suspected. Establishing reasonable cut-off points for serum ferritin (e.g., >800 ng/mL) and TSAT (e.g., >50%) can guide clinicians on when to escalate to MRI, ensuring that its use is both practical and targeted for high-risk patients. This approach would help balance the need for advanced diagnostics with the realities of resource limitations.

Personalized chelation therapy

In BTM, chelation therapy is tailored to the individual’s iron burden, transfusion history, and tolerance to chelating agents [20]. The most commonly used chelators include deferasirox, deferoxamine, and deferiprone, each with specific indications based on patient needs and the degree of iron overload [21]. Personalized therapy ensures patients receive the appropriate chelation to prevent iron-related complications while minimizing side effects [1].

Although chelation therapy is not routinely used in KF, there is growing recognition of its potential in patients with significant iron overload due to repeated transfusions or excessive iron supplementation [22]. A lesson from BTM is the importance of individualized treatment plans. In KF patients, careful titration of iron doses based on regular monitoring and the possible use of chelation therapy in selected cases could help manage iron overload more effectively [23]. Therefore, titrating iron supplementation in patients with excessive iron stores could prevent further accumulation.

Furthermore, the management of iron overload differs significantly between transfusion-dependent thalassemia and hemodialysis patients. In thalassemia, iron chelation therapy is essential to address transfusional iron overload due to chronic anemia from ineffective erythropoiesis. In contrast, hemodialysis patients face cumulative blood loss from the dialysis process, frequent blood sampling, and uremic enteropathy [24]. Rather than iron chelation, reducing or halting iron infusions effectively addresses iron accumulation in these patients [24].

Multidisciplinary care

Effective management of iron overload in BTM involves a multidisciplinary approach. Hematologists, cardiologists, hepatologists, and endocrinologists collaborate to monitor and manage iron-related complications in various organs [25]. This collaborative approach ensures that patients receive comprehensive care tailored to their needs.

Similarly, KF patients with iron overload could benefit from multidisciplinary care involving nephrologists, hematologists, and cardiologists. Since iron overload can affect multiple organs, including the heart and liver, a team-based approach would facilitate comprehensive monitoring and management. For example, routine cardiac evaluations using MRI T2* could help detect early cardiac iron deposition, allowing timely intervention to prevent heart failure [9].

Minimizing Iron Intake

In BTM, limiting the number of transfusions and optimizing chelation therapy are vital strategies to minimize iron overload [26]. While transfusions are necessary to maintain adequate hemoglobin levels in BTM, reducing the frequency of transfusions when medically appropriate can limit iron intake. Additionally, optimizing the efficiency of chelation therapy ensures that excess iron is effectively removed [1].

Iron supplementation is often necessary to manage anemia in KF, but over-supplementation or inappropriate dosing can lead to iron overload. A lesson from BTM is the importance of balancing iron intake with the patient’s actual needs. KF patients, especially those on ESAs, require careful monitoring to avoid excessive iron supplementation. A more judicious use of iron therapy, tailored to the individual’s iron stores and response to treatment, could help reduce the risk of overload.

Patient education and compliance

Patient education plays a crucial role in managing iron overload in BTM. Patients are educated about the importance of regular monitoring and adherence to chelation therapy, which can be burdensome but is essential for preventing complications [27]. Similarly, educating patients about the risks of iron overload and the importance of adhering to prescribed treatments can improve compliance and outcomes in KF. Patients with KF often undergo complex treatment regimens, including iron therapy, ESAs, and dialysis, making understanding each component's role in their care essential. Educating patients about the signs of iron overload, such as unexplained fatigue or organ dysfunction, can also empower them to seek timely medical advice.

Preventive strategies for managing iron overload

Incorporating preventive measures to manage iron overload in KF patients receiving intravenous (IV) iron supplementation is essential. One practical strategy is to assess serum ferritin and TSAT levels before each iron infusion to prevent excessive iron accumulation. Calculating individualized iron requirements based on the patient's current iron status and therapeutic needs, rather than administering a fixed dose, could also optimize iron therapy. This tailored approach mirrors practices in beta-thalassemia management, where personalized chelation regimens based on iron burden and transfusion history have improved outcomes [28]. A similar individualized strategy in KF could minimize iron overload while ensuring effective anemia management.

## Conclusions

Managing iron overload in BTM provides valuable lessons for managing iron overload in KF patients. Early detection using non-invasive imaging, personalized chelation therapy, multidisciplinary care, and patient education are all strategies that can be adapted from BTM management to improve outcomes in KF patients. As KF patients face the challenge of balancing anemia management with the risk of iron overload, these lessons offer practical insights for optimizing care and preventing iron-related complications.

## References

[1] Cappellini MD, Porter JB, Viprakasit V, Taher AT (2018). A paradigm shift on beta-thalassaemia treatment: how will we manage this old disease with new therapies?. Blood Rev.

[2] Shah FT, Porter JB, Sadasivam N (2022). Guidelines for the monitoring and management of iron overload in patients with haemoglobinopathies and rare anaemias. Br J Haematol.

[3] Nashwan AJ, Yassin MA, Abd-Alrazaq A, Shuweihdi F, Othman M, Abdul Rahim HF, Shraim M (2023). Hepatic and cardiac iron overload quantified by magnetic resonance imaging in patients on hemodialysis: a systematic review and meta-analysis. Hemodial Int.

[4] Nashwan AJ, Abuawwad MT, Jaradat JH, Ibraheem A, Yassin MA, Taha MJ (2024). Prevalence of iron overload in patients with chronic kidney disease on peritoneal dialysis: a scoping review. Health Sci Rep.

[5] Shah FT, Sayani F, Trompeter S, Drasar E, Piga A (2019). Challenges of blood transfusions in β-thalassemia. Blood Rev.

[6] Aydinok Y, Porter JB, Piga A (2015). Prevalence and distribution of iron overload in patients with transfusion-dependent anemias differs across geographic regions: results from the CORDELIA study. Eur J Haematol.

[7] Taher AT, Saliba AN (2017). Iron overload in thalassemia: different organs at different rates. Hematology Am Soc Hematol Educ Program.

[8] Nashwan AJ, Athikkal SA, Rao AG (2024). Point sheer wave elastography (pSWE) and transient elastography (TE) for quantifying iron overload and liver fibrosis in beta-thalassemia patients. J Med Surg Public Health.

[9] Anderson LJ, Holden S, Davis B (2001). Cardiovascular T2-star (T2*) magnetic resonance for the early diagnosis of myocardial iron overload. Eur Heart J.

[10] Macdougall IC, Bircher AJ, Eckardt KU (2016). Iron management in chronic kidney disease: conclusions from a "Kidney Disease: Improving Global Outcomes" (KDIGO) Controversies Conference. Kidney Int.

[11] Babitt JL, Lin HY (2012). Mechanisms of anemia in CKD. J Am Soc Nephrol.

[12] Kalantar-Zadeh K, Streja E, Miller JE, Nissenson AR (2009). Intravenous iron versus erythropoiesis-stimulating agents: friends or foes in treating chronic kidney disease anemia?. Adv Chronic Kidney Dis.

[13] Gaweda AE, Ginzburg YZ, Chait Y, Germain MJ, Aronoff GR, Rachmilewitz E (2015). Iron dosing in kidney disease: inconsistency of evidence and clinical practice. Nephrol Dial Transplant.

[14] Nashwan AJ, Yassin MA, Mohamed Ibrahim MI, Abdul Rahim HF, Shraim M (2022). Iron overload in chronic kidney disease: less ferritin, more T2(*) MRI. Front Med (Lausanne).

[15] Yassin MA, Soliman AT, De Sanctis V (2018). Statural growth and prevalence of endocrinopathies in relation to liver iron content (LIC) in adult patients with beta thalassemia major (BTM) and sickle cell disease (SCD). Acta Biomed.

[16] Yassin M, Soliman A, De Sanctis V (2017). Liver iron content (LIC) in adults with sickle cell disease (SCD): correlation with serum ferritin and liver enzymes concentrations in transfusion dependent (TD-SCD) and non-transfusion dependent (NT-SCD) patients. Mediterr J Hematol Infect Dis.

[17] Nashwan AJ, Alkhawaldeh IM, Shaheen N (2023). Using artificial intelligence to improve body iron quantification: a scoping review. Blood Rev.

[18] Aslan E, Luo JW, Lesage A (2021). MRI-based R2* mapping in patients with suspected or known iron overload. Abdom Radiol (NY).

[19] Grant C, Juarez D, Pakbaz Z (2023). Iron overload in dialysis patients: serum ferritin underestimates liver iron measured by MRI. Blood.

[20] Yassin MA, Soliman AT, De Sanctis V (2018). Jadenu(®)substituting Exjade(®) in iron overloaded β-thalassemia major (BTM) patients: a preliminary report of the effects on the tolerability, serum ferritin level, liver iron concentration and biochemical profiles. Mediterr J Hematol Infect Dis.

[21] Entezari S, Haghi SM, Norouzkhani N (2022). Iron chelators in treatment of iron overload. J Toxicol.

[22] Nashwan AJ, Yassin MA (2023). Deferasirox in patients with chronic kidney disease: assessing the potential benefits and challenges. J Blood Med.

[23] Lee KH, Ho Y, Tarng DC (2021). Iron therapy in chronic kidney disease: days of future past. Int J Mol Sci.

[24] Rostoker G, Vaziri ND, Fishbane S (2016). Iatrogenic iron overload in dialysis patients at the beginning of the 21st century. Drugs.

[25] Pinto VM, Forni GL (2020). Management of iron overload in beta-thalassemia patients: clinical practice update based on case series. Int J Mol Sci.

[26] Saliba AN, Harb AR, Taher AT (2015). Iron chelation therapy in transfusion-dependent thalassemia patients: current strategies and future directions. J Blood Med.

[27] Yadav PK, Singh AK (2022). A review of iron overload in beta-thalassemia major, and a discussion on alternative potent iron chelation targets. Plasmatology.

[28] Taher AT, Bou-Fakhredin R, Kattamis A, Viprakasit V, Cappellini MD (2021). Improving outcomes and quality of life for patients with transfusion-dependent β-thalassemia: recommendations for best clinical practice and the use of novel treatment strategies. Expert Rev Hematol.

